# Systemic embolization in infective endocarditis

**DOI:** 10.1007/s12055-023-01616-2

**Published:** 2023-11-06

**Authors:** Henrik Agerup Kildahl, Evelyn Lauvstad Brenne, Håvard Dalen, Alexander Wahba

**Affiliations:** 1 Department of Cardiothoracic Surgery, St Olavs Hospital, Trondheim, Norway; 2 Department of Circulation and Medical Imaging, Faculty of Medicine and Health Sciences, Norwegian University of Science and Technology, Box 8905, 7491 Trondheim, Norway; 3 Department of Cardiology, St Olavs Hospital, Trondheim, Norway

**Keywords:** Systemic embolization, Infective endocarditis, Cardiothoracic surgery

## Abstract

Embolism is a common complication in infective endocarditis which may lead to serious complications, such as stroke, intestinal ischemia, and peripheral embolization. A comprehensive literature search was performed and the registry at our centre, including 390 cases of infective endocarditis, diagnosed between 2010 and 2020, was investigated. Large registries show that 20–40% of patients with infective endocarditis (IE) are affected by embolism. In many instances, embolism is present already at the time of diagnosis. The rate of embolism during the hospital stay in our data was 11%. However, only 2% developed clinical embolism during or following surgery. According to recent guidelines, previous embolism, and the presence of vegetations > 10 mm present an indication for surgical treatment. Routine imaging revealed non-symptomatic cerebral embolism in 8.5% of surgical patients. However, it is not clear whether detection of non-symptomatic embolism and consecutive surgical treatment improves the prognosis of infective endocarditis.

## Introduction

Infective endocarditis (IE) of the heart valves may lead to structural damage of the affected heart valve with loss of valve tissue and ensuing dysfunction, mainly valve incompetence and consequentially heart failure. According to the guidelines, an indication for valve surgery in the left sided valve infected endocarditis may result from heart failure, uncontrolled infection and for prevention of embolism [1]. Prevention of embolism refers to the fact that bacterial activity commonly leads to a buildup of vegetations, consisting of fibrin, platelets, and bacteria, held together by agglutinating antibodies [2]. These vegetations are fragile structures that may be dislodged by the blood stream resulting in peripheral and cerebral emboli [3]. There is still a lack of knowledge on how frequently embolism occurs. However, recent publications suggest that embolism is a common occurrence in IE, which unfortunately can result in serious complications [3]. In the European Infective Endocarditis Registry (EURO-ENDO) study, embolic events were the most frequent complications in IE [3]. With a history of recurrent systemic embolization, surgical treatment should be considered [4]. In this overview, the authors will summarize the literature and discuss the frequency of embolization during the course of the disease, the resulting morbidity, and possibilities for prevention. We will also present recent results from our own investigation.

## Methods

### Study design

This review uses findings from a Master of Science Project conducted at our centre involving patients treated for infective endocarditis in the period 2010 to 2020. We further aim to summarize other available literature regarding the incidence, distribution, and prevention of emboli in infective endocarditis and combine the two in this review.

### Data collection

The protocol from the study conducted at our centre involved collecting data from a hospital register of patients with infective endocarditis. They were further identified using a Hospital Trust-specific patient identification number. The necessary information collected from the patient’s electronic journal was then structured in a de-identified research database. All parts of the study were approved by the Regional Committees for Medical and Health Research Ethics. The research board at the Clinic of Cardiology approved the use of clinical hospital data regarding this study.

### Literature search

Searching for literature in PubMed, January 10th, 2022, using the search terms ‘cardiopulmonary bypass’ AND ‘infectious endocarditis’ AND (‘stroke OR embolization’) we identified 118 manuscripts. The summaries were reviewed and evaluated by two observers, and 30 manuscripts were judged as relevant by at least one observer. They were evaluated comprehensively in a second step leaving 16 manuscripts as relevant, whereof nine were hospital registry data, five were case series or case presentations, and three were reviews.

### Patient selection

Patients diagnosed with infective endocarditis at St. Olavs University hospital in the period between 2010 and 2020 were included. Infective endocarditis was defined when patients fulfilled the criteria for ‘definite’ or ‘possible’ endocarditis according to the Duke criteria.

Patients were identified by the search for International Classification of Diseases 10^th^ revision (ICD-10) diagnosis code “I33”. All patients with any ICD-10 “I33.*” diagnosis were included in the database for validation and data collection.

After a review of the medical records, 81 hospital stays were not classified as definite or possible endocarditis according to the Duke criteria, seven of the admissions were in individuals < 18 years, four were treated at other hospitals and four were pacemaker infections and not infective endocarditis. Finally, after the exclusion of these 98 admissions, a total of 416 admissions and 390 individual patients were included in the analyses.

A total of 148 (38%) patients were treated with cardiac surgery with the use of cardiopulmonary bypass circulation and 242 (62%) patients were conservatively treated. The management of all patients was according to the present European Society of Cardiology Guidelines.

### Study procedures

From the study at our centre, we studied the associations of cerebral hemorrhages, cerebral infarctions, and non-cerebral embolies with time to surgery. In addition, the authors have reviewed the available literature about emboli in infective endocarditis to summarize what is currently known regarding emboli in infective endocarditis, with focus on incidence, prevalence, and measures of prevention.

### Statistical methods

Data from the study at our centre were presented according to their distribution and analysed accordingly. Linear regression analyses were used to test associations in the case of scalar-dependent variables. Time to surgery was not normally distributed and therefore this variable was log-transformed when studying associations of cerebral hemorrhages, cerebral infarctions and non-cerebral embolies with time to surgery. This did not alter the result and non-transformed results are presented here in this review. Survival data were presented as Kaplan–Meier plots. Mantel-Cox log-rank statistics were used to compare event-free survival between groups tests (unadjusted analyses) and Cox proportional hazard analyses were performed to compare event-free survival between the groups adjusted for age. All analyses were performed by the IBM SPSS Statistics software (SPSS Inc, Chicago, IL, USA; version 27).

### Study limitations

As all decisions regarding management and treatment of the patients, they are solely based on clinical decisions creating important limitations to the infective endocarditis study conducted at our centre. Firstly, this may limit the generalizability, as management of patients may vary between cardiac centers, even though the decisions are based upon current guidelines. Secondly diagnostic imaging was performed only in 1/3 of patients, based on the clinical decision. Thus, the incidence of ischemic and hemorrhagic events may be underreported.

### Definitions

From the study conducted at our centre, complications were defined as death or any cerebral hemorrhage or any cerebral or non-cerebral emboli and compared between the surgical and the non-surgical group. In this analysis, we used the date of cardiac surgery as the entering point in the surgical group and the day of admission in the non-surgical group. Any complication diagnosed before day 30 was counted.

## Incidence of embolization

The incidence of symptomatic embolic events, particularly with cerebral manifestations, is high and the current literature suggests that 20–40% of patients with IE are affected.

The largest publication, covering embolism in endocarditis is the EURO-ENDO study [3], a multicenter study including 156 centres from 40 countries and 3116 patients, with definite or possible IE. The registry data show that embolic events were the most common complication of endocarditis, occurring in over 40% of cases [3]. A noticeable 25% of patients had already symptomatic emboli upon admittance, and the rate of new emboli during the hospital stay was reported to be 20%, despite adequate treatment.

Another large survey, the Euro Heart Survey, is a multicenter study with 92 centres from 25 countries. 5001 patients with valvular heart disease (VHD), of whom 159 patients had active IE, were enrolled. In this population, 27% of patients had at least 1 embolic episode on admission [5].

These findings were supported by the International Collaboration on Endocarditis-Prospective Cohort Study (ICE-PCS), a multicenter study with 58 centres from 25 countries and 2781 included patients. In this study, stroke was reported in 17% and embolization (other than stroke) in 23% of patients, with staphylococcus aureus being the most common agent [6]. Thuny et al. 2007 [7] showed a presence of one or more embolic event in 45% of patients admitted with definitive IE. Di Salvo et al. 2001 [8] showed that one or more embolic event was present in 37% of patients admitted with definite infective endocarditis.

**Fig. 1:**
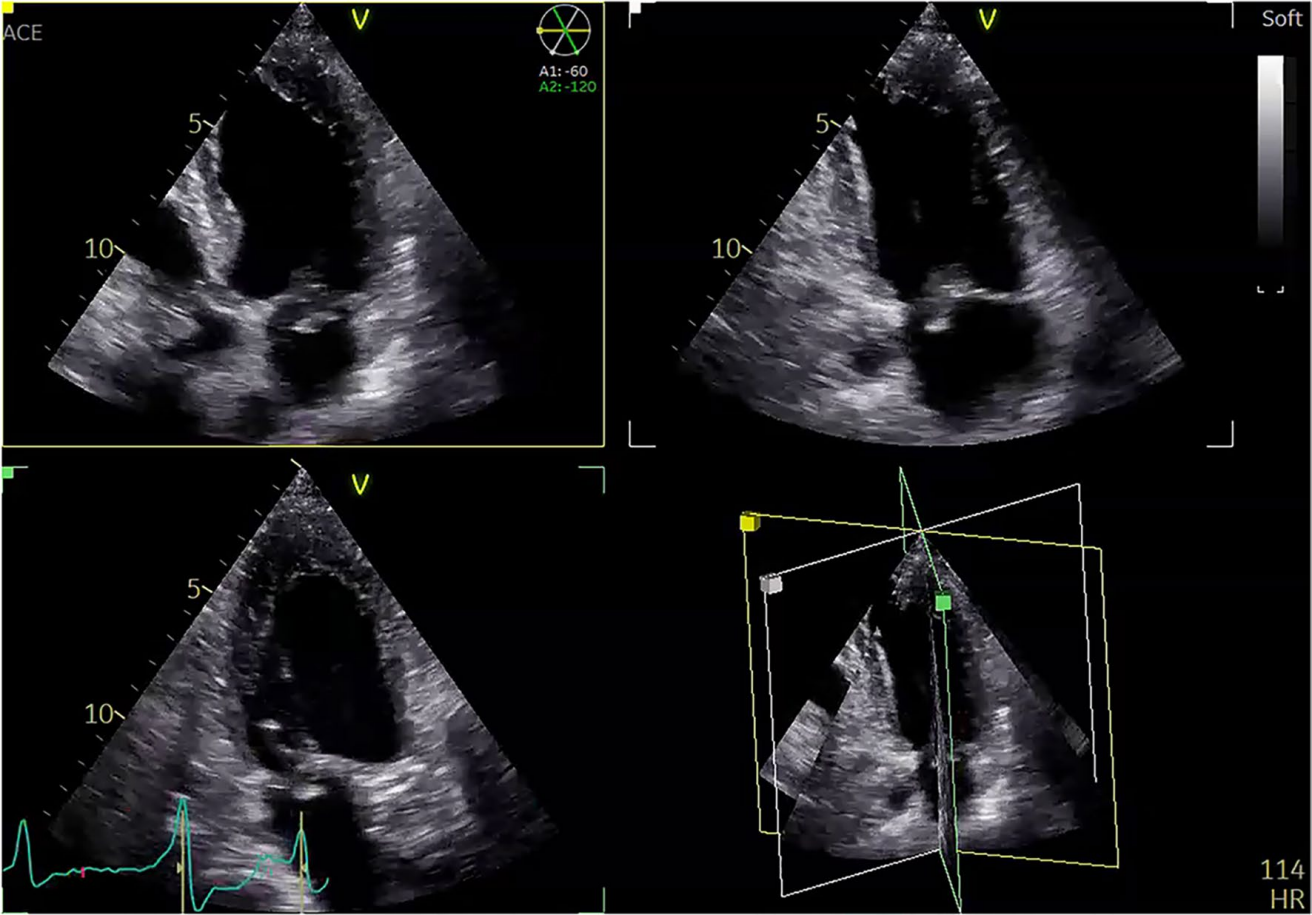
Transthoracal echocardiographic imaging of a mitral valve endocarditis

Results from the published literature are in line with our own findings in the cohort of patients treated for infective endocarditis at St. Olavs University Hospital. Symptomatic embolic events were common in our population, present in 166 of 390 patients (43%). In the conservatively treated group, the prevalence of any symptomatic emboli was 98 (41%), compared to 68 (46%) in the surgical treatment group. The prevalence of emboli was further divided into cerebral or non-cerebral (Table 1). Both the conservatively and surgically treated group were managed according to current guidelines at our center (Fig. 1).

**Table 1:** Cerebral complications in IE patients according to if surgery was performed or not (numbers from MSc thesis Cardiac surgery with cardiopulmonary bypass and the associations with stroke, bleeding and embolic events in infective endocarditis by Evelyn Lauvstad Brenne)

	Conservative treatment	Surgical treatment	*p*-value
N	242	148	
Any emboli or stroke, n (%)	98 (41%)	68 (46%)	0.31
Cerebral infarctions, n (%)	50 (21%)	44 (30%)	0.045
Cerebral hemorrhages, n (%)	8 (3.3%)	3 (2%)	0.54
Hemorrhagic transition of cerebral infarctions, n (%)	7 (2.9%)	2 (1.4%)	0.35
Any non-cerebral emboli, n (%)	62 (26%)	40 (27%)	0.76

Definition of terms used in Table 1. ‘Emboli’ includes both cerebral and non-cerebral as well as left-sided and right-sided events. ‘Stroke’ includes both infarctions and any cerebral hemorrhage. ‘Cerebral hemorrhage’ includes intra-cerebral (parenchymal) and other hemorrhages as well as hemorrhagic transitions of cerebral infarctions. Non-cerebral embolies include embolies to the column, skin, liver, spleen, lung, and others (e.g., gastrointestinal, pancreas)

*Abbreviation*: *N* numbers

## Cerebral embolization

In the study from our hospital, cerebral infarctions were common both in patients treated conservatively and surgically, with prevalence of 21% and 30% respectively, which is in line with findings in the EURO-ENDO study, where cerebral embolism was found in 25% during the course of the disease.

In the Euro Heart Survey cerebral embolic events were found in 15% of patients on admission, which is similar to the 13% found in the EURO-ENDO study. In our data, the presence of cerebral embolic events was somewhat higher, with 18% in the conservatively treated group and 23% in the surgical group (Table 2).

**Table 2:** Timing and group allocation of cerebral events in IE patients (numbers from MSc thesis Cardiac surgery with cardiopulmonary bypass and the associations with stroke, bleeding and embolic events in infective endocarditis Evelyn Lauvstad Brenne)

	Conservative treatment	Surgical treatment	*p*-value
Timing of cerebral infarction events			
Present at admission	41 (18%)	34 (23%)	0.22
During hospital stay	12 (5.3%)	16 (11%)	0.04
After discharge	5 (2.2%)	2 (1.4%)	0.71
Timing of cerebral hemorrhagic events			
Present at admission	10 (4.4%)	2 (1.4%)	0.14
During hospital stay	2 (0.9%)	1 (0.7%)	1.00
After discharge	2 (0.9%)	0	0.52
Timing of any embolic events			
Present at admission	79 (34.5%)	52 (35.4%)	0.76
During hospital stay	25 (11%)	20 (14%)	0.44
After discharge	8 (3.5%)	3 (2.0%)	0.54

Data are presented as numbers (%). *P*-values for the difference between groups are shown. Hemorrhagic transition of ischemic strokes is included both in cerebral infarctions and cerebral hemorrhage

*Abbreviations*: *CPB* cardiopulmonary bypass circulation, *n* numbers

The EURO-ENDO results confirm that a significant number of embolic events occurred after admission, with an incidence of 20% during hospitalization. In our dataset, this was 11% for the conservative treatment and 14% for the surgical treated group. In our material, patients who underwent cardiac surgery for IE were twice as likely to have a cerebral infarction during the hospital stay compared to the conservatively treated patients. However, most of the cerebral infarctions in the surgical group (13 of 16) occurred before surgery, with median time from embolic event to surgery being 8 days. Only 3 patients (2%) developed embolism during or following surgery. Apparently, the occurrence of cerebral events during treatment influenced the decision to perform surgery.

Cerebral embolization has been shown to be related to in-hospital mortality [3, 9]. The EURO-ENDO study states that cerebral embolism was the cause of death in 12% of patients dying from IE in the study.

In addition to symptomatic cerebral embolism, non-symptomatic embolism may be found on cerebral imaging. Misfield et al. [9] reported an incidence of non-symptomatic cerebral infarction in 8.5% discovered by routine computerized tomography (CT) scan in addition to 24% (*n* = 375) of patients with symptomatic cerebral emboli. This was further supported by a later study that found a higher detection of emboli when routine neuroimaging was performed [10]. The significance of such findings was underlined by Misfeld et al., who reported a significantly impaired long-term survival, with no difference between symptomatic and asymptomatic cerebral embolization. The 5-year survival was 46% in patients with cerebral embolism, compared with 57% in patients without cerebral embolism [9]. The authors thus recommend routine cerebral imaging in all IE patients.

## Non-cerebral embolization

Emboli to non-cerebral locations are less common than cerebral events. However, it is fairly common that patients with cerebral emboli also experience emboli to other organs, in particular to the spleen [9, 11]. One study found that 49% of patients with cerebral emboli also experienced non-cerebral emboli [9]. The Euro Heart Survey showed cerebral emboli in 24 patients, pulmonary, abdominal and miscellaneous embolies in six, two and nine patients, respectively [5].

In the EURO-ENDO study, symptomatic non-cerebral embolism on admission was most commonly found in the lungs and abdominal organs and rarely to the extremities. In our own study, non-cerebral emboli were found in one quarter of patients during the course of the disease. IE of right-sided valves is associated with pulmonary embolism, which may be a clinically relevant feature associated with increased mortality [1, 3].

**Fig. 2:**
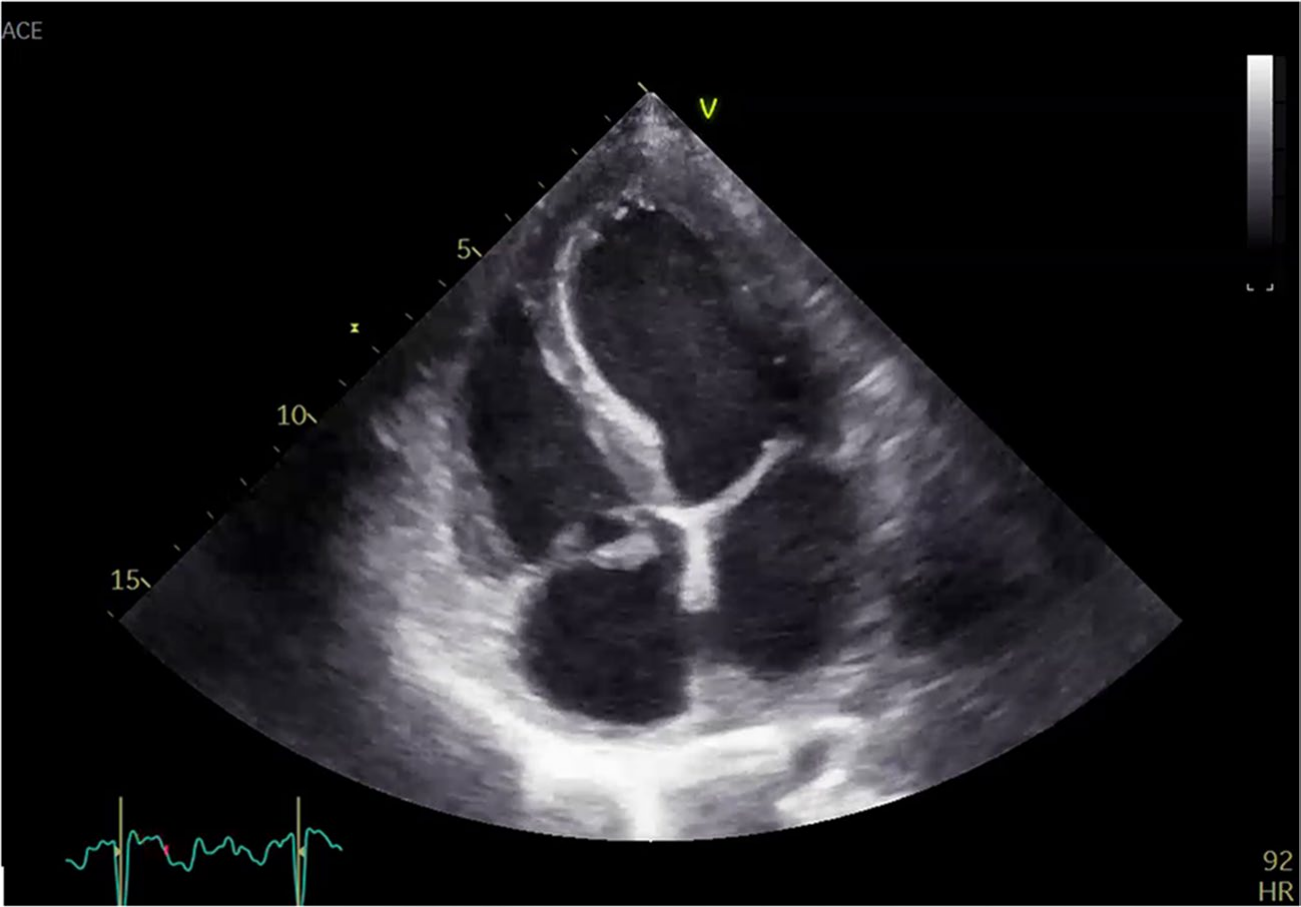
Transthoracal echocardiography of a tricuspid valve endocarditis

Embolism to abdominal organs may be clinically silent. However, acute intestinal artery occlusion due to emboli in the setting of infective endocarditis presents a rare surgical emergency [12]. Endocarditis should be considered as a possible origin for emboli in such a case (Fig. 2).

## Risk factors for embolism

Several predisposing factors have been discussed in the literature. The EURO-ENDO study found evidence for the following risk factors: age, previous pulmonary embolism, heart failure, statins, previous Vitamin K antagonist (VKA) therapy, aortic, tricuspid IE or device related infective endocarditis, pulmonary endocarditis, intracardiac defibrillator/pacemaker endocarditis, vegetation presence and size, positive blood cultures, and *Staphylococcus aureus* infection [3]. In this study, there were positive blood cultures in 79.0% of patients, with staphylococci being the most isolated bacteria (44.1%). Enterococci and oral streptococci were found in 15.8% and 12.4% of cultures respectively. At our centre, we saw the same microorganisms, with staphylococci in 37.4% and streptococci in 34% of cases. Both the Euro Heart Survey and other publications have stated that size and mobility of vegetations are an important predictor of risk for recurrent embolism [5], with a substantially greater risk for embolic events when the diameter of the vegetation is > 10 mm [13]. Monitoring of vegetations by cardiac ultrasound may be particularly helpful to predict recurrent embolism [14]. Very large vegetations are considered to be particularly hazardous [15, 16]. Vegetation size should be considered when reflecting on surgical treatment, due to the risk of embolization [1, 5, 16].

## Imaging

There is a discrepancy in the frequency of imaging performed for emboli in infective endocarditis, as well as the choice of imaging methods. As previously mentioned, Misfeld et al. [9] made an interesting finding that a considerable number of patients with IE had asymptomatic cerebral infarctions detected on routine computed tomography (CT) scans. In the EURO-ENDO study, several imaging techniques were listed in the diagnostic process of IE including echocardiography, multislice CT (MSCT), magnetic resonance imaging (MRI), nuclear imaging (positron emission tomography (PET)-CT and leukocyte scintigraphy). However, there is a lack of information regarding the preferred methods for detecting emboli. In our dataset, 33–34% of all patients were examined with either CT, MRI or ultrasound for detection of cerebral infarctions/hemorrhages and non-cerebral embolies. CT was the preferred method when embolization was suspected, but only used when emboli was clinically suspected (Table 3).

**Table 3:** Cerebral and non-cerebral diagnostic imaging for evaluation of infective endocarditis complications (numbers from MSc thesis Cardiac surgery with cardiopulmonary bypass and the associations with stroke, bleeding and embolic events in infective endocarditis by Evelyn Lauvstad Brenne)

	Conservative treatment	Surgical treatment	*p*-value
N	242	148	
Any CT, MRI, or ultrasound, n (%)	79 (33%)	50 (34%)	0.03
Cerebral CT, n (%)	11 (5%)	7 (5%)	0.49
Cerebral MRI, n (%)	26 (11%)	24 (16%)	< 0.01
Non-cerebral CT, n (%)	32 (13%)	23 (16%)	0.07
Non-cerebral MRI, n (%)	21 (9%)	7 (5%)	0.58
Ultrasound for non-cerebral embolies, n (%)	3 (1%)	1 (1%)	1.00
Positron emission tomography, n (%)	26 (11%)	6 (4%)	0.01

*Abbreviations: CT* computed tomography, *MRI* magnetic resonance imaging, *N* numbers

PET was rarely used in our hospital, and it was used even less frequently in the surgical group. This was due to lack of availability of PET at our centre until recently.

The EURO-ENDO survey found that the choice of imaging modality differed significantly between regions of the world, with PET being more common in Western Europe. In Northern Europe, fluorodeoxyglucose positron emission tomography (FDG PET)-CT scan was performed in 25.6% of all cases. Overall, FDG PET-CT scan was used in 16.6% of the patients in the EURO-ENDO survey and 38.8% (*n* = 201) had extra-cardiac uptake, with the most common locations being lung (27%), spine (22%), spleen (20%), bowel (19%) and liver with 5%.

Tornos et al. 2005 [5] mostly used CT, US and arteriography in the search for embolic events; however, radiologic examinations in the search for embolic events where not used particularly often, with CT examinations being used in only a quarter of the patients included.

## Prevention of embolic events

Early implementation of appropriate treatment is considered to be the most important means to prevent embolic events or prevent recurrent embolism [1]. This refers both to antibiotic treatment and to surgical treatment. Very large vegetations may in specific circumstances justify surgery in its own right [14, 16]. Although some studies recommend routine imaging to discover emboli in the setting of infective endocarditis [3, 9, 10], the consequences of positive findings are discussed controversially in the literature [1]. In most cases, the decision to operate will depend on finding a balance between possible advantages of early surgery, the expected course of the recovery with conservative treatment, and the risk of surgery. In the majority of cases, the indication for surgery will be based on several factors [5]. The risk for embolic events has been shown to be highest in the early phase of IE [17]. ENDO-EURO states that one of the key reasons for withholding surgery, although indicated, was death before surgery could be performed (23% of cases), further underlining the importance of an early evaluation of surgical intervention by the Heart Team.

In our study, 67 patients had at least one preoperative embolic event and 52 (69%) of all 75 embolic events and 34 (65%) of 52 cerebral embolic events were already present upon admission. The median time from admission to surgery in the studied population at our centre was 5.4 days. We found no significant association of the incidence of cerebral hemorrhage/infarction or non-cerebral emboly with whether or when cardiac surgery was performed.

## Conclusion

Embolic events are common complications in both surgically and conservatively treated IE patients. According to recent guidelines, surgery is indicated both in the event of uncontrolled infection and for the prevention of recurrent embolism. In our material, surgical patients were affected by cerebral hemorrhages/infarctions or non-cerebral emboli at similar frequencies compared to conservatively treated patients. Our findings suggest that the surgical management of infective endocarditis at St. Olavs University hospital, from 2010 to 2020, which was performed in 38% out of 390 patients after a median of 5 days, is within published results. The EURO-ENDO states that there still is a poor prognosis in patients with infective endocarditis, with the possible need of a more aggressive treatment. Other publications made similar suggestions [3, 18]. However, whether detection of clinically silent embolism by routine imaging in asymptomatic patients, and consecutive more frequent surgical treatment, results in improved outcomes is a matter of debate. However, other reasons for an early surgical approach may be found in subgroups of patients with aggressive bacteria or when valve repair, instead of replacement, is anticipated [19].

## Data Availability

The data supporting the findings are found within this review.
